# Opening avenue for the targeted treatment of lung cancer using xanthohumol loaded nanostructured lipid carriers

**DOI:** 10.17179/excli2024-7656

**Published:** 2024-08-28

**Authors:** Shubham Singh, Sangeeta Saxena, Himani Sharma, Gaurav Gupta, Kamal Dua, Sachin Kumar Singh

**Affiliations:** 1 School of Biosciences and Engineering, Lovely Professional University, Phagwara, Punjab, India; 2 Department of Biotechnology, Babasaheb Bhimrao Ambedkar University, Lucknow, India; 3 Center of Medical and Bio-allied Health Sciences Research, Ajman University, Ajman, United Arab Emirates; 4 Center for Research Impact & Outcome, Chitkara College of Pharmacy, Chitkara University, Rajpura, Punjab 140401, India; 5 Discipline of Pharmacy, Graduate School of Health, University of Technology Sydney, Ultimo, NSW 2007, Australia; 6 Faculty of Health, Australian Research Center in Complementary and Integrative Medicine, University of Technology Sydney, Ultimo, NSW 2007, Australia; 7 School of Pharmaceutical Sciences, Lovely Professional University, Phagwara, Punjab, India

## ⁯⁯⁯⁯

According to Global Observatory Cancer (GLOBOCAN) 2020, the number of new cases of cancer diagnosed in 2020 was about 19.3 million and among them, 10.0 million people died. GLOBOCAN predicts that the number of cancer cases will increase up to 28.4 million by 2040 (Sung et al., 2021[9]). Lung cancer (LC) has a higher prevalence rate and mortality. The estimated number of LC up to 2020 was 2.2 million and 2 million people died among them. LC is mainly caused due to smoking, exposure to second-hand smoke, radiation, exposure to radon gas, and exposure to asbestos or other carcinogenic agents. There are two types of LC, small cell lung cancer (SCLC) and non-small cell lung cancer (NSCLC). SCLC accounts for the prevalence rate of 10-15 % and causes early widespread metastasis. It is most common among heavy smokers. NSCLC accounts for ~85 % of all LC cases which are given in Rosell and Karachaliou (2016[8]). NSCLC affects active as well as passive smokers. It mainly arises in bronchi and peripheral lung tissue. Metastasis is a major cause of death in patients with malignant tumors, accounting for ~90 % of cancer-associated deaths (Bielenberg and Zetter, 2015[2]; Nemeth et al., 2003[7]). NSCLC has been classified into three categories including adenocarcinoma, squamous cell carcinoma and large cell carcinoma. Adenocarcinoma is the most common type of LC, about 40 % of NSCLC. It begins in the epithelial cells that line the outside of the lungs. Squamous cell carcinoma starts in squamous cells. Squamous cells are flat cells that line the inside of lung. About 25-30 % of all NSCLC cases are squamous cell LC. Large cell carcinoma is the least common type of NSCLC. It accounts for about 10 % of NSCLC.

The currently available conventional methods that have been used for the treatment of LC include surgery, chemotherapy, radiotherapy, immunotherapy and various nanoparticle mediated formulation targeting the LC. Chemotherapy utilizes synthetic drugs such as cisplatin, carboplatin, paclitaxel, etoposide, and docetaxel. While radiotherapy utilizes gamma radiation. Chemotherapy and radiotherapy have many sides effect like hair loss, mouth soreness, nausea and vomiting. They also affect the normal cell along with cancer cells. Chemotherapy can damage some healthy cells in the body, such as blood cells, skin cells and cells in the stomach. While surgery for cancer patients is highly painful, immunotherapy is too costly to be achieved by patients for the treatment of LC, hence there is an immediate requirement for safe, effective and cost-effective therapy. Therefore, the use of plant-derived bioactives that are secondary metabolites has gained attention in treating LC owing to better safety profile and their inhibitory action on multiple signaling pathways of LC. Among them, Xanthohumol (Xn) and its formulations have been found effective in treating cancer.

Xanthohumol (Xn) is a prenylated chalconoid that is found in the female inflorescences of *Humulus lupulus*. It is also known as hops. The structure of Xn was first identified by Verzele et al. (1957[10]). In recent years, much research has been carried out to harness the anticancer potential of Xn. These studies indicated that Xn is effective against leukemia (Wang et al., 2020[11]), NSCLC (Gao et al., 2020[3]), and breast cancer (Gieroba et al., 2020[4]). Some studies revealed that Xn possesses good antioxidant, anti-inflammatory, antibacterial, antiviral, antifungal, and anti-plasmodial activity (Liu et al., 2015[6]).

To trigger the cell cycle, Xn activates p53 and increases p21 protein levels. Apoptosis in cancer cells was aided by the treatment with Xn because it decreased the expression of Bcl-2 and Survivin. Xn was discovered to exert its inhibitory effect on the growth and proliferation of cancer by modulating numerous signaling pathways, including MAPK, EGFR/AKT, WNT/-CATENIN, ERK, NF-κB, STAT3 as well as various proteins, including caspases, Bcl-2, CyclinD1, Oxidative markers, and tumor suppressor gene (Girisa et al., 2021[5]). The mechanism of action of Xn is shown in the supplementary information (Supplementary Figure 1).

Despite having the wonderful anticancer potential of Xn, it has certain physiochemical/biopharmaceutical challenges that impede its bioavailability. These challenges include poor aqueous solubility and poor absorption from biological membranes including GIT. Delivering to the target site is challenging because of its high hydrophobic nature that leads to poor internalization. Thus, Xn fails to produce an effective therapeutic response to the target site of LC. Therefore, to overcome the challenges Xn loaded NLCs can be formulated to boost bioavailability and give an efficient therapeutic effect against the lung carcinoma cells to address these issues. NLCs are composed of a solid lipid and liquid lipid blend along with surfactants and co-surfactants. This leads to an imperfect matrix structure and less dense lipid packaging. It generates space to incorporate drugs in the matrix. Therefore, NLCs have a higher drug encapsulation efficiency (EE) and stability during long-term storage (Almurshedi et al., 2021[1]). These NLCs, when given through the intranasal route to the lungs, will reach the tumor cells and elicit their action against LC. Hence, intranasal administration of Xn-loaded NLCs could offer effective treatment against LC. The mechanism of delivery of Xn via NLCs into lung at lung cells is shown in the supplementary information (Supplementary Figure 2). 

## Notes

Kamal Dua and Sachin Kumar Singh (School of Pharmaceutical Sciences, Lovely Professional University, Phagwara-144411, Punjab, India; Tel. +91-9888720835, E-mail: singhsachin23@gmail.com) contributed equally as corresponding author.

## Conflict of interest

The authors declare no conflict of interest.

## Supplementary Material

Supplementary information
