# Supplementary material for: Opening avenue for the targeted treatment of lung cancer using xanthohumol loaded nanostructured lipid carriers
Source: EXCLI J. 2024 Aug 28;23:1068–70. doi: 10.17179/excli2024-7656 (PMC11483262; doi:10.17179/excli2024-7656)
Supplement: Supplementary information [file EXCLI-23-1068-s-001.pdf]

EXCLI Journal 2024;23:1068-1070 – ISSN 1611-2156
Received: January 24, 2024, accepted: April 26, 2024, published: August 28, 2024

Supplementary information to:

Letter to the editor:

OPENING AVENUE FOR THE TARGETED TREATMENT OF LUNG CANCER USING XANTHOHUMOL LOADED NANOSTRUCTURED LIPID CARRIERS

Shubham Singh[a], Sangeeta Saxena[b], Himani Sharma[a], Gaurav Gupta[c,d], Kamal Dua[e,f,*], Sachin Kumar Singh[f,g,*]

[a] School of Biosciences and Engineering, Lovely Professional University, Phagwara, Punjab, India
[b] Department of Biotechnology, Babasaheb Bhimrao Ambedkar University, Lucknow, India
[c] Center of Medical and Bio-allied Health Sciences Research, Ajman University, Ajman, United Arab Emirates
[d] Center for Research Impact & Outcome, Chitkara College of Pharmacy, Chitkara University, Rajpura, Punjab 140401, India
[e] Discipline of Pharmacy, Graduate School of Health, University of Technology Sydney, Ultimo, NSW 2007, Australia
[f] Faculty of Health, Australian Research Center in Complementary and Integrative Medicine, University of Technology Sydney, Ultimo, NSW 2007, Australia
[g] School of Pharmaceutical Sciences, Lovely Professional University, Phagwara, Punjab, India

* **Corresponding authors:** Sachin Kumar Singh, School of Pharmaceutical Sciences, Lovely Professional University, Phagwara-144411, Punjab, India. Tel. +91-9888720835; E-mail: singhsachin23@gmail.com
Kamal Dua, Discipline of Pharmacy, Graduate School of Health, University of Technology Sydney, P.O. Box 123 Broadway, Ultimo, NSW, 2007, Australia.
E-mail: Kamal.Dua@uts.edu.au

https://dx.doi.org/10.17179/excli2024-7656

This is an Open Access article distributed under the terms of the Creative Commons Attribution License (http://creativecommons.org/licenses/by/4.0/).

EXCLI Journal 2024;23:1068-1070 – ISSN 1611-2156
Received: January 24, 2024, accepted: April 26, 2024, published: August 28, 2024

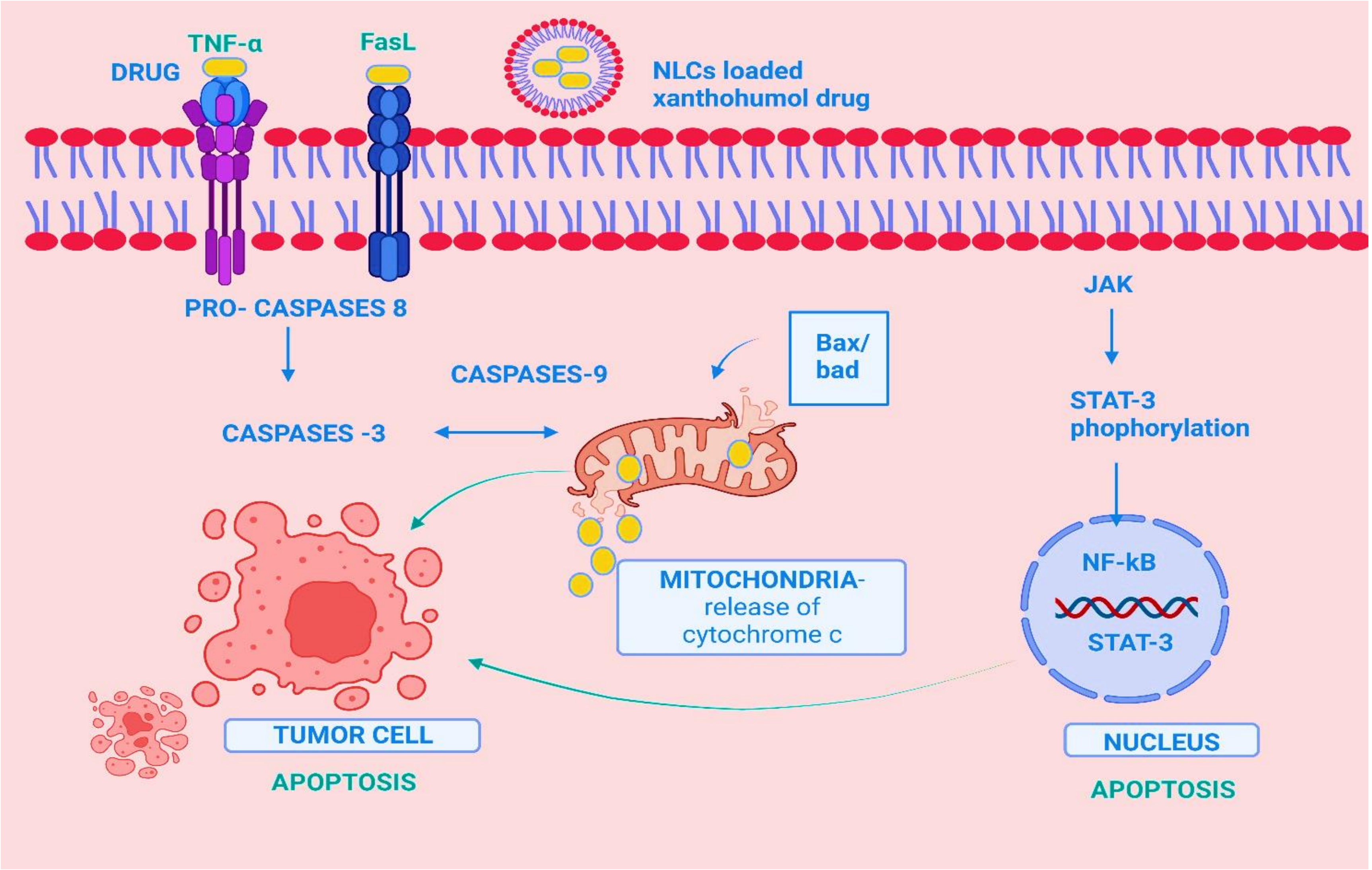

Supplementary Figure 1: Apoptosis showing in tumor cells by the action of NLCs loaded with xanthohumol drug

EXCLI Journal 2024;23:1068-1070 – ISSN 1611-2156
Received: January 24, 2024, accepted: April 26, 2024, published: August 28, 2024

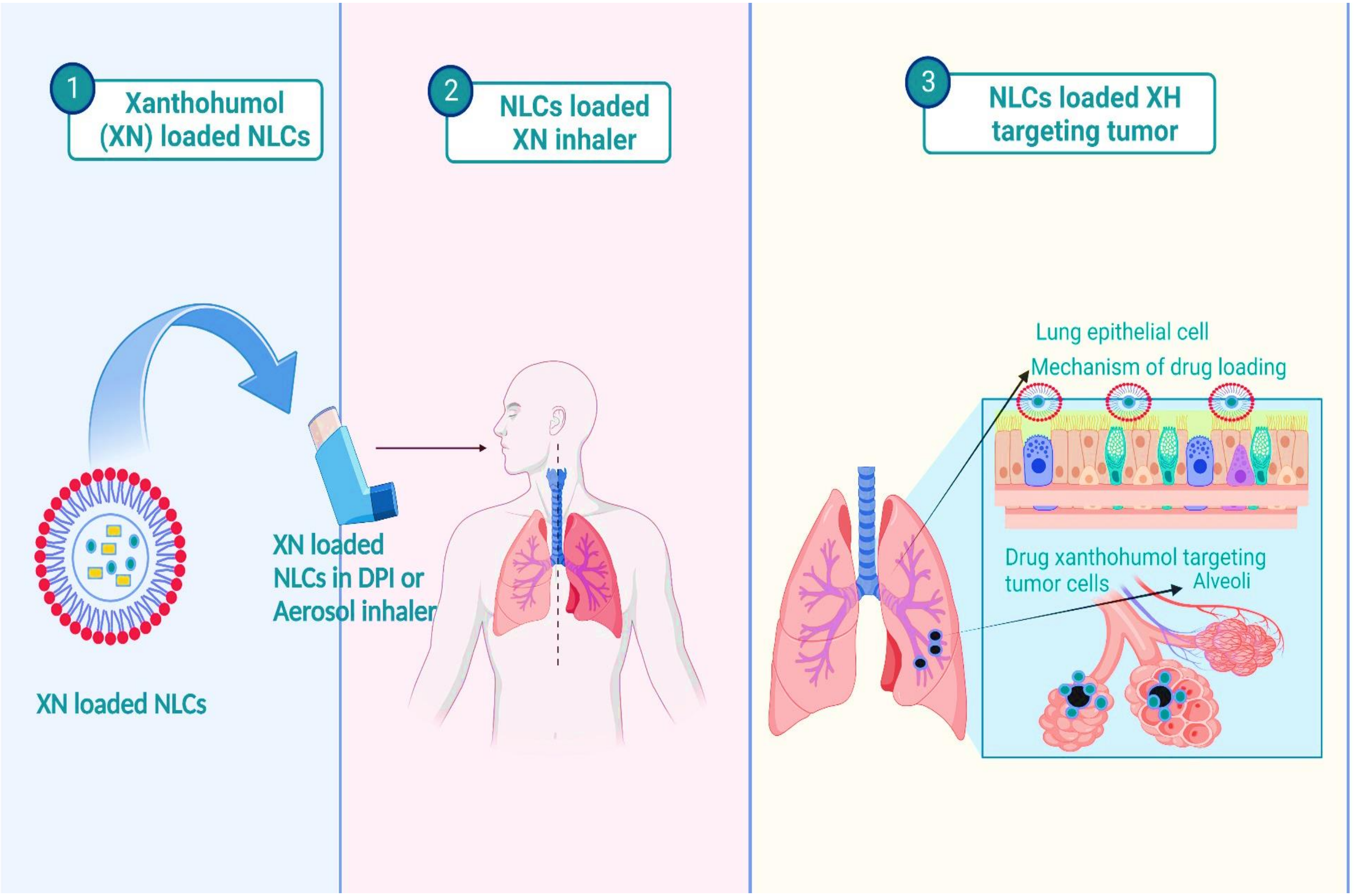

Supplementary Figure 2: Mechanism involved in pulmonary delivery of drug loaded nanostructure lipid carriers NLCs
